# Supplementary material for: Fronto-Parietal Gray Matter Volume Loss Is Associated with Decreased Working Memory Performance in Adolescents with a First Episode of Psychosis
Source: J Clin Med. 2021 Aug 31;10(17):3929. doi: 10.3390/jcm10173929 (PMC8432087; doi:10.3390/jcm10173929)
Supplement: Supplementary file 1 [file jcm-10-03929-s001.zip › jcm-1302258-supplementary.pdf]

Supplementary Table S1. Comparison of Clinical and functional characteristics of completers and non-completers# at baseline

	Completers (n=33)		Non-Completers (n=77)		*Test**	*p value*
	**Mean**	**DS**	**Mean**	**DS**		
Age	15.85	1.46	15.39	1.872	*t=1.253*	*p=0.213*
Gender (Female/Male)	9/24		27/50		*X^2=0.425*	*p=0.509* Fisher's exact test
Race (Caucasians/Others)	29/4		65/12		*X^2=0.637*	*p=0.773* Fisher's exact test
Years of education	8.15	2.412	8.12	2.439	*t=0.068*	*p=0.946*
Estimate IQ	81.21	16.435	78.35	22.281	*t=0.66*	*p=0.511*
Panss Positive	24.97	5.987	23.43	6.693	*t=1.141*	*p=0.256*
Panss Negative	18.82	8.921	20.53	8.792	*t=-0.933*	*p=0.353*
Panss General	45.03	13.047	45.1	9.389	*t=-0.033*	*p=0.973*
Panss Total	88.82	23.732	89.06	18.384	*t=-0.059*	*p=0.953*
Gaf	34.64	15.556	33	14.594	*t=0.528*	*p=0.598*

Abbreviations: GAF, Children's Global Assessment of Functioning; MRI, magnetic resonance imaging; PANSS, Positive and Negative Syndrome Scale. IQ=Intellectual Quotient

Non-completers are participants for whom we do not have a baseline or follow-up cognitive or magnetic resonance imaging data or from whom the data were not good enough to be used for the study.

*Student's t or X2 test comparative analyses between completers and non-completers at baseline

Supplementary Table S2. Associations and predictive value of change in brain volume measures over change in sustained attention and executive function at two-year follow-up in EOP (Initial prediction models for non-significant backwards regression analyses are displayed – see methods).

**DV*:** CHANGE IN SUSTAINED ATTENTION OVER TIME					
**PREDICTORS**: Change in Frontal Left GM Volume, age, months of inter-scan interval and interscan ICV change.					
**Model Summary**	**R**	**R^2**	**Adjusted R^2**	**Standard Error of the Estimate**	
	0.324	0.105	-0.230	0.63128	
**Model 4**	**B**	**Standard Error**	**Beta**	**t**	**p**
**Change in Frontal Left GM Volume**	0.002	0.022	0.020	0.100	0.921
**Age**	0.007	0.006	0.215	1.185	0.246
**Months of inter-scan interval**	-0.036	0.056	-0.126	-0.647	0.523
**Interscan ICV change**	-0.145	0.110	-0.240	-1.321	0.197
**PREDICTORS**: Change in Frontal Right GM Volume, age, months of inter-scan interval and interscan ICV change.					
**Model Summary**	**R**	**R^2**	**Adjusted R^2**	**Standard Error of the Estimate**	
	0.324	0.105	-0.230	0.63138	
**Model 4**	**B**	**Standard Error**	**Beta**	**T**	**p**
**Change in Frontal Right GM Volume**	0.001	0.022	0.006	0.029	0.977
**Age**	0.007	0.006	0.212	1.184	0.247
**Months of inter-scan interval**	-0.035	0.055	-0.12	-0.633	0.532
**Interscan ICV change**	-0.146	0.110	-0.241	-1.328	0.195
**PREDICTORS**: Change in Parietal Left GM Volume, age, months of inter-scan interval and interscan ICV change.					
**Model Summary**	**R**	**R^2**	**Adjusted R^2**	**Standard Error of the Estimate**	
	0.356	0.127	0.002	0.62351	

Model 4	B	Standard Error	Beta	T	p
**Change in Parietal Left GM Volume**	0.022	0.026	0.159	0.844	0.406
**Age**	0.008	0.006	0.241	1.334	0.193
**Months of inter-scan interval**	-0.047	0.054	-0.163	-0.872	0.390
**Interscan ICV change**	-0.142	0.108	-0.235	-1.309	0.201
**PREDICTORS**: Change in Parietal Right GM Volume, age, months of inter-scan interval and interscan ICV change.					
**Model Summary**	**R**	**R^2**	**Adjusted R^2**	**Standard Error of the Estimate**	
	0.396	0.157	0.036	0.61277	
**Model 4**	**B**	**Standard Error**	**Beta**	**T**	**p**
**Change in Parietal Right GM Volume**	0.037	0.028	0.233	1.314	0.199
**Age**	0.008	0.006	0.240	1.369	0.182
**Months of inter-scan interval**	-0.045	0.052	-0.155	-0.869	0.392
**Interscan ICV change**	-0.147	0.106	-0.243	-1.382	0.178
**DV*:** CHANGE IN EXECUTIVE FUNCTION OVER TIME					
**PREDICTORS**: Change in Frontal Left GM Volume, age, months of inter-scan interval and interscan ICV change.					
**Model Summary**	**R**	**R^2**	**Adjusted R^2**	**Standard Error of the Estimate**	
	0.354	0.125	0.000	0.56795	
**Model 4**	**B**	**Standard Error**	**Beta**	**T**	**p**
**Change in Frontal Left GM Volume**	0.019	0.020	0.188	0.971	0.340
**Age**	-0.004	0.006	-0.124	-0.689	0.497
**Months of inter-scan interval**	-0.092	0.051	-0.347	-1.807	0.081
**Interscan ICV change**	-0.026	0.099	-0.048	-0.268	0.791

**PREDICTORS**: Change in Frontal Right GM Volume, age, months of inter-scan interval and interscan ICV change.					
**Model Summary**	**R**	**R^2**	**Adjusted R^2**	**Standard Error of the Estimate**	
	0.334	0.112	-0.015	0.57224	
**Model 4**	**B**	**Standard Error**	**Beta**	**T**	**p**
**Change in Frontal Right GM Volume**	0.014	0.020	0.134	0.714	0.481
**Age**	-0.005	0.005	-0.150	-0.841	0.407
**Months of inter-scan interval**	-0.085	0.050	-0.321	-1.695	0.101
**Interscan ICV change**	-0.030	0.099	-0.054	-0.300	0.766
**PREDICTORS**: Change in Parietal Left GM Volume, age, months of inter-scan interval and interscan ICV change.					
**Model Summary**	**R**	**R^2**	**Adjusted R^2**	**Standard Error of the Estimate**	
	0.311	0.097	-0.032	0.57705	
**Model 4**	**B**	**Standard Error**	**Beta**	**t**	**p**
**Change in Parietal Left GM Volume**	-0.005	0.024	-0.036	-0.190	0.851
**Age**	-0.005	0.006	-0.157	-0.857	0.399
**Months of inter-scan interval**	-0.071	0.050	-0.270	-1.425	0.165
**Interscan ICV change**	-0.034	0.100	-0.061	-0.336	0.739
**PREDICTORS**: Change in Parietal Right GM Volume, age, months of inter-scan interval and interscan ICV change.					
**Model Summary**	**R**	**R^2**	**Adjusted R^2**	**Standard Error of the Estimate**	
	0.309	0.096	-0.034	0.57742	
**Model 4**	**B**	**Standard Error**	**Beta**	**T**	**p**
**Change in Parietal Right GM Volume**	0	0.027	-0.003	-0.018	0.986
**Age**	-0.005	0.006	-0.151	-0.832	0.412
**Months of inter-scan interval**	-0.074	0.049	-0.280	-1.517	0.141

**Interscan ICV change**	-0.033	0.100	-0.060	-0.328	0.746

*Dependent Variable (DV), Grey Matter (GM)

Supplementary Table S3. Pearson correlations between changes in symptomatology and antipsychotic medication and changes in cognitive performance and brain volume in patients with a first episode of psychosis.

	**Possitive Panss**		**Negative Panss**		**General Panss**		**Total Panss**		**Antipsychotic Medication**	
	n=33		n=33		n=33		n=33		n=33	
	***r**	***P***	***r**	***p***	***r**	***P***	***r**	***p***	***r**	***p***
**Sustained Attention**	-0.226	0.206	0.094	0.601	-0.019	0.917	-0.035	0.848	0.191	0.302
**Working Memory**	0.181	0.314	0.184	0.305	0.015	0.933	0.128	0.477	-0.094	0.614
**Executive Function**	0.018	0.920	-0.139	0.439	0.001	0.996	-0.049	0.788	0.105	0.572
**Frontal Left Grey Matter Volume**	0.046	0.797	0.024	0.894	-0.036	0.840	0.003	0.987	-0.078	0.668
**Frontal Right Grey Matter Volume**	0.010	0.956	-0.045	0.802	-0.119	0.509	0.077	0.670	-0.094	0.603

**Parietal Left Grey Matter volume**	-0.147	0.416	-0.023	0.898	-0.056	0.757	-0.078	0.666	- 0.020	0.911
**Parietal Right Grey Matter volume**	-0.169	0.348	-0.037	0.838	-0.034	0.850	-0.078	0.667	0.055	0.762

* r = Pearson correlation coefficient. Significance set at $p \leq 0.05$;
